# Associations of umbilical cord vitamin D levels with childhood cardiometabolic risks: a longitudinal mother–child study

**DOI:** 10.3389/fnut.2026.1777909

**Published:** 2026-04-13

**Authors:** Di Mao, Hanbin Wu, Lai-Yuk Yuen, Chung-Shun Ho, Claudia Ha-Ting Tam, Michael Ho-Ming Chan, William L. Lowe, Ronald Ching-Wan Ma, Chi-Chiu Wang, Wing-Hung Tam

**Affiliations:** 1 Department of Obstetrics and Gynecology, The Chinese University of Hong Kong, Hong Kong, Hong Kong SAR, China; 2 Department of Chemical Pathology, The Chinese University of Hong Kong, Hong Kong, Hong Kong SAR, China; 3 Department of Medicine and Therapeutics, The Chinese University of Hong Kong, Hong Kong, Hong Kong SAR, China; 4 Department of Medicine, Northwestern University Feinberg School of Medicine, Chicago, IL, United States; 5 Li Ka Shing Institute of Health Sciences, The Chinese University of Hong Kong, Hong Kong, Hong Kong SAR, China; 6 Hong Kong Institute of Diabetes and Obesity, The Chinese University of Hong Kong, Hong Kong, Hong Kong SAR, China; 7 School of Biomedical Sciences, The Chinese University of Hong Kong, Hong Kong, Hong Kong SAR, China

**Keywords:** cardiometabolic risk, childhood, pregnancy, umbilical cord, vitamin D

## Abstract

**Background:**

Vitamin D inadequacy is globally prevalent among pregnant women, but its impact on offspring cardiometabolic risks remains inconclusive. This study aimed to evaluate associations between in utero vitamin D status and childhood cardiometabolic risk factors.

**Methods:**

In the original Hyperglycaemia and Adverse Pregnancy Outcome (HAPO) Study, all pregnant women booked for antenatal care, except teenage pregnancy, plan to delivery at another hospital, uncertain last menstrual period and no ultrasonographic estimated gestational age, inability to complete the oral glucose tolerance test (OGTT), multiple pregnancy, conception by assisted reproduction, glucose testing before recruitment, or diagnosis of diabetes during or before the current pregnancy and required medication. Archived maternal serum samples from 24 to 32 gestational weeks and umbilical cord serum samples at birth in the original HAPO Study at the Hong Kong Centre were assayed for total 25-hydroxyvitamin D [25(OH)D] levels by using liquid chromatography-tandem mass spectroscopy. Children’s clinical and biochemistry parameters were collected at the HAPO Follow-Up Study at around age 7, including anthropometry, blood pressure (BP), carotid-femoral pulse wave velocity (cfPWV), fasting lipid profile, plasma glucose (PG), and insulin levels at OGTT. Linear regression analyses were used to evaluate the associations of in-utero 25(OH)D levels with children’s cardiometabolic risk factors.

**Findings:**

There was no association between maternal serum total 25(OH)D level in 24–32 weeks of gestation and childhood cardiometabolic risk factors. In contrast, umbilical cord serum total 25(OH)D level at birth was negatively associated with offspring diastolic BP, cfPWV, and fasting PG at around age 7, whereas no significant results were observed in other cardiometabolic risk factors. Every 1-SD (18.0 nmol/L) increase in the umbilical cord serum total 25(OH)D level was independently associated with a reduction in diastolic BP, cfPWV, and fasting PG by 0.73 mmHg (95% CI [−1.44, −0.02], *p* = 0.044), 0.08 m/s ([−0.14, −0.03], *p* = 0.004), and 0.04 mmol/L ([−0.07, −0.01], *p* = 0.017), respectively. However, only associations with cfPWV and fasting PG remained significant after correction for multiple testing.

**Interpretation:**

A low umbilical cord serum 25(OH)D level at birth, but not maternal serum 25(OH)D in 24–32 weeks of gestation, was associated with higher childhood diastolic BP, arterial stiffness, and fasting PG levels. Although maternal total 25(OH)D levels between 24 and 32 gestational weeks were not associated with childhood cardiometabolic risks, our findings indicated that neonatal vitamin D status at birth may be relevant to childhood vascular and glucose metabolic health. Given the observational study design and modest effect size, these findings should be interpreted cautiously and warrant further investigations.

## Introduction

Serving as a crucial nutrient for maintaining bone health and preventing a spectrum of chronic diseases (1), vitamin D deficiency is becoming a severe public health issue, whether in Asia or worldwide (2–4). With the increasing global prevalence of vitamin D deficiency in adults, extensive evidence has shown that low vitamin D is associated with increased risks of hypertension, diabetes, and cardiovascular disease events later in life (5–7). Importantly, vitamin D deficiency is common during pregnancy and also common early in the life course, and over 50% of pregnant women and neonates have vitamin D deficiency (8). Within the Western Pacific Region, various risk factors such as variations in latitude and diet significantly influence the maternal and neonatal vitamin D status. Consequently, the prevalence of vitamin D deficiency in pregnant women and neonates fluctuates widely, with higher rates observed in Asian countries (9–11).

Fetal 25-hydroxyvitamin D [25(OH)D] levels are primarily dependent on the maternal vitamin D status. It has been reported that low maternal vitamin D level during pregnancy was associated with greater placental vitamin D transfer, suggesting a possible protective mechanism for fetal development (12, 13). Thus far, several studies have explored the association of low in utero vitamin D status, including maternal and umbilical cord serum 25(OH)D, with offspring cardiometabolic risk factors, such as blood pressure (BP) and glucose metabolism from birth to young adulthood (14–20). Identifying the risk factor before birth or in early-life is critical, as childhood cardiometabolic manifestations are known to extend into adulthood, increasing the future development and burden of type 2 diabetes mellitus, cardiovascular disease, and premature mortality (21–23). However, the associations between intrauterine hypovitaminosis D and childhood cardiometabolic risk factors remain inconclusive, likely due to heterogeneity in the timing of blood samples for 25(OH)D measurement, ranging from first trimesters of pregnancy to delivery; and the use of different vitamin D assay methods, using immunoassays or liquid chromatography–tandem mass spectrometry (LC–MS/MS). Given these sampling time and methodological challenges, the associations of hypovitaminosis D in utero with the offspring cardiometabolic risk factors are inconsistent and warrant further investigation.

This study aimed to assess the association of maternal serum total 25(OH)D levels, collected at 24–32 weeks of gestation, and umbilical cord serum total 25(OH)D levels at birth, as measured by LC–MS/MS, with children’s cardiometabolic risks at around 7 years of age.

## Materials and methods

### Study cohort and setting

This study included mother–child pairs who were ethnic Chinese and participated in the Hyperglycaemia and Adverse Pregnancy Outcome (HAPO) Follow-Up Study in 2009–2013 at the Hong Kong Centre. Details of the original HAPO Study and the HAPO Follow-Up Study were previously described (24, 25). In the original HAPO Study, all pregnant women booked for antenatal care in the study centre were eligible to participate unless they met an exclusion criteria, namely, teenage pregnancy younger than 18 years of age, plan to undergo delivery at another hospital, an uncertain date of last menstrual period and no ultrasonographic estimate of gestational age between 6 and 24 weeks, inability to complete the oral glucose tolerance test (OGTT) within 32 weeks of gestation, multiple pregnancy, conception by means of assisted reproduction, glucose testing before recruitment, diagnosis of diabetes during the current pregnancy, or diagnosis of diabetes before the current pregnancy and requiring treatment with medication. All pregnant women with a singleton pregnancy underwent a 75-g OGTT at 24–32 weeks of gestation, and the results were blinded to the participants and the clinical team. The adiposity rebound occurs around age 7, which is critically associated with future adiposity in later life, at around 16 years of age (26). Mothers were invited to attend a follow-up assessment along with their children born from the index pregnancy, at an average of 7 years after the original HAPO Study.

All mothers provided a written consent form to participate in the original HAPO study, and its ancillary studies, while written informed consent of their offspring was obtained from parents or legal guardians at the HAPO Follow-up study to allow archived serum to be used for biochemical analysis in future studies, as described previously (25, 27). The study was approved by the Joint Chinese University of Hong Kong-New Territories East Cluster Clinical Research Ethics Committee (CREC Ref. No.:2008.017 and 2019.572).

Maternal demographic data, including parity, education, and smoking, and children’s medical history, family history, and physical activity at the follow-up assessment at 7 years of age, were recorded using structured questionnaires as previously described (25). Anthropometric parameters included body height, weight, waist circumference, hip circumference, skinfold thickness, BP, and carotid-femoral pulse wave velocity (cfPWV). Children’s standing body height without shoes was measured to the nearest 0.1 cm using a Harpenden stadiometer (Holtain Ltd., Crymych, U. K.); their body weight (with light clothing) was measured to the nearest 0.1 kg using a Tanita physician digital scale (model no. TBF 410; Tanita Corp., Tokyo, Japan). Children’s BMI was calculated as body weight in kilograms divided by height in meters squared. Waist circumference, at the midpoint between the lower ridge of the ribs and the top of the iliac crest, was measured to the nearest 0.1 cm using a nonelastic flexible tape. Hip circumference was measured at the broadest circumference below the waist. We measured skinfold thickness at four sites on the right side (biceps, triceps, subscapular, and suprailiac) using a Holtain Tanner/Whitehouse skinfold caliper (Holtain Ltd.). Children’s BP was measured three times in the nondominant arm using an Omron T5 BP monitor (Omron Healthcare Co. Ltd., Kyoto, Japan) at 1-min intervals. Age-, sex-, and height-specific percentiles for systolic BP and diastolic BP were computed using data released from the National High Blood Pressure Education Program Working Group on High Blood Pressure in Children and Adolescents (28). Prehypertension/hypertension was defined as systolic or diastolic BP percentile above the 90th percentile. Children’s cfPWV was measured twice using the Sphygmo Cor apparatus (SphygmoCor1 Px, AtCor Medical, Australia) as previously described (29). cfPWV was computed as the pulse wave travel distance divided by the transit time, where the travel distance was the distance between 2 measuring sites (carotid and femoral) and transit time was the time it takes for the arterial pulse to travel from the proximal to the distal measuring site (30). All participants were advised to fast overnight for ≥ 8 h on the day before the evaluation of arterial stiffness.

### Biochemical test

After an overnight fast, all children had an OGTT at five time points after receiving a glucose load of 1.75 g/kg body weight, or a 75-g glucose load if they weighed ≥42.8 kg, with blood taken at 0, 15, 30, 60, and 120 min to measure plasma glucose (PG) and insulin levels. PG was measured with the hexokinase method, using an automated analyser (Hitachi 911; Boehringer Mannheim, Mannheim, Germany). Impaired fasting glucose (IFG, fasting PG between 5.6 and <7.0 mmol/L), impaired glucose tolerance (IGT, 2-h PG between 7.8 and <11.1 mmol/L), and diabetes (Fasting PG ≥ 7.0 mmol/L or 2-h PG ≥ 11.1 mmol/L) were defined according to the American Diabetes Association guidelines (31). Abnormal glucose tolerance was defined as the presence of IFG, IGT, or diabetes. Plasma insulin levels were analyzed using an immunoassay analyzer (Immulite 1,000 Immunoassay System; Siemens, Munich, Germany). Insulin sensitivity was calculated using the Matsuda insulin sensitivity index (ISI), calculated by (10,000/square root of [PG _0-min_ × Insulin _0-min_] × [PG _mean_ × Insulin _mean_]); the Homeostasis model assessment (HOMA) of insulin resistance was also used to assess the insulin sensitivity. Pancreatic *β*-cell function was determined using the HOMA of β-cell function. The insulinogenic index, a surrogate for first-phase insulin secretion of the OGTT, was estimated using the formula [(I^30^ – I^0^) (pmol/L) ÷ (G^30^ – G^0^) (mmol/L)], where G^0^ and G^30^ were the fasting and 30-min PG levels, and I^0^ and I^30^ were the fasting and 30-min insulin levels, respectively.

Children’s fasting blood was also collected to determine serum 25(OH)D level. Serum total 25(OH)D level, which represents the vitamin D status, was computed as the sum of serum 25(OH)D_2_ and 25(OH)D_3_. Serum 25(OH)D_2_ and 25(OH)D_3_ levels were measured by the LC–MS/MS method that enabled the separation of 3-epi-25(OH)D_3_, the biologically inactive form, from the vitamin D metabolites as previously described (27). Fasting plasma triglyceride, HDL cholesterol, and LDL cholesterol levels were measured with enzymatic methods using a DP Modular Analytics system (Roche Diagnostics, Indianapolis, IN). Detailed laboratory assays were previously described (25).

### Statistical analysis

Although no universally accepted umbilical cord serum-specific thresholds for vitamin D are currently available, several studies have defined neonatal vitamin D deficiency as < 50 or < 30 nmol/L, and severe deficiency as < 25 or 12.5 nmol/L, using umbilical cord serum 25(OH)D concentrations, respectively (32–36). To ensure consistency and comparability between maternal and umbilical cord measurements, the same thresholds were applied to both maternal and umbilical cord serum total 25(OH)D concentration in the current study. Following our previous study (13), maternal and umbilical cord serum vitamin D statuses were categorized into severe deficient (< 25 nmol/L), deficient (25- < 50 nmol/L), insufficient (50- < 75 nmol/L), and sufficient (≥75 nmol/L), which is strictly based on the Endocrine Society clinical practice guidelines (37). Ambient solar radiation data in mid-gestation and at birth were collected from the Hong Kong Observatory website (https://www.hko.gov.hk/en/index.html), as previously described (13).

Continuous data were expressed as mean ± standard deviation (SD), while categorical data were expressed as counts (percentage). Comparisons across maternal or umbilical cord vitamin D categories were performed primarily for descriptive and exploratory purposes. For continuous variables, between-group differences were compared by ANOVA; when distributions were non-normal, the Kruskal-Wallis test was used. For categorical variables, chi-square tests or Fisher’s exact tests were used, as appropriate. For continuous outcomes, P for trend was obtained from linear regression models in which the ordered categories of serum total 25(OH)D were entered as a continuous variable. The scatter plot was used to depict the linear associations of maternal and umbilical cord serum total 25(OH)D levels with children’s vitamin D, BP, cfPWV, and glucose levels at around 7 years of age.

Univariable and multivariable linear regression analyses were used to explore the association of maternal and umbilical cord serum 25(OH)D levels with children’s BP, cfPWV, and glucose levels. Potential confounders were forced into the multivariable models. Notably, children’s serum vitamin D concentration at around age 7 was handled according to its hypothesized causal role in maternal serum vitamin D and umbilical cord vitamin D models. If it was considered to lie on the causal pathway between the exposure and childhood cardiometabolic risk factors, then it was treated as a potential mediator and was not included in the covariate adjustment. If, however, it was not considered to lie on the causal pathway for a given exposure-outcome model but was considered a variable associated with both the exposure and outcome, then it was treated as a potential confounder and adjusted for in the model. To account for the multiple testing across the cardiometabolic risk factors, the Benjamini-Hochberg correction was used to control the false discovery rate (FDR) at 5% involved in investigating the associations of in utero vitamin D and childhood cardiometabolic risks.

Additionally, we also explored nonlinear associations between in-utero vitamin D levels and children’s BP, cfPWV, and glucose levels by using polynomial regression models with two degrees, such as a quadratic model, and the *F* test to determine the statistical significance of the quadratic model. The R^2^ change was also used to assess the superiority of the quadratic model over the linear model, by using an *F* test, and a significant R^2^ change implies a better association in the quadratic model.

All analyses were performed using the R 4.5.1 software (www.r-project.org; R Foundation for Statistical Computing, Vienna, Austria) with the analysis package stats (version 4.5.1). *p* < 0.05 was considered statistically significant.

## Results

### Baseline characteristics

Of the 973 mother–child pairs returned for HAPO follow-up assessment between 2009 and 2013, maternal serum vitamin D data were available in 897 (92.2%) children, and umbilical cord serum vitamin D data were available in 807 (82.9%) children. Comparisons of baseline characteristics between those who attended the follow-up and those lost to follow-up are shown in Supplementary Table 1. Maternal and neonatal baseline characteristics and children’s follow-up characteristics are presented in Supplementary Table 2.

### Distribution of children’s cardiometabolic risk factors by maternal serum vitamin D levels at the time of OGTT

**Figure 1:**
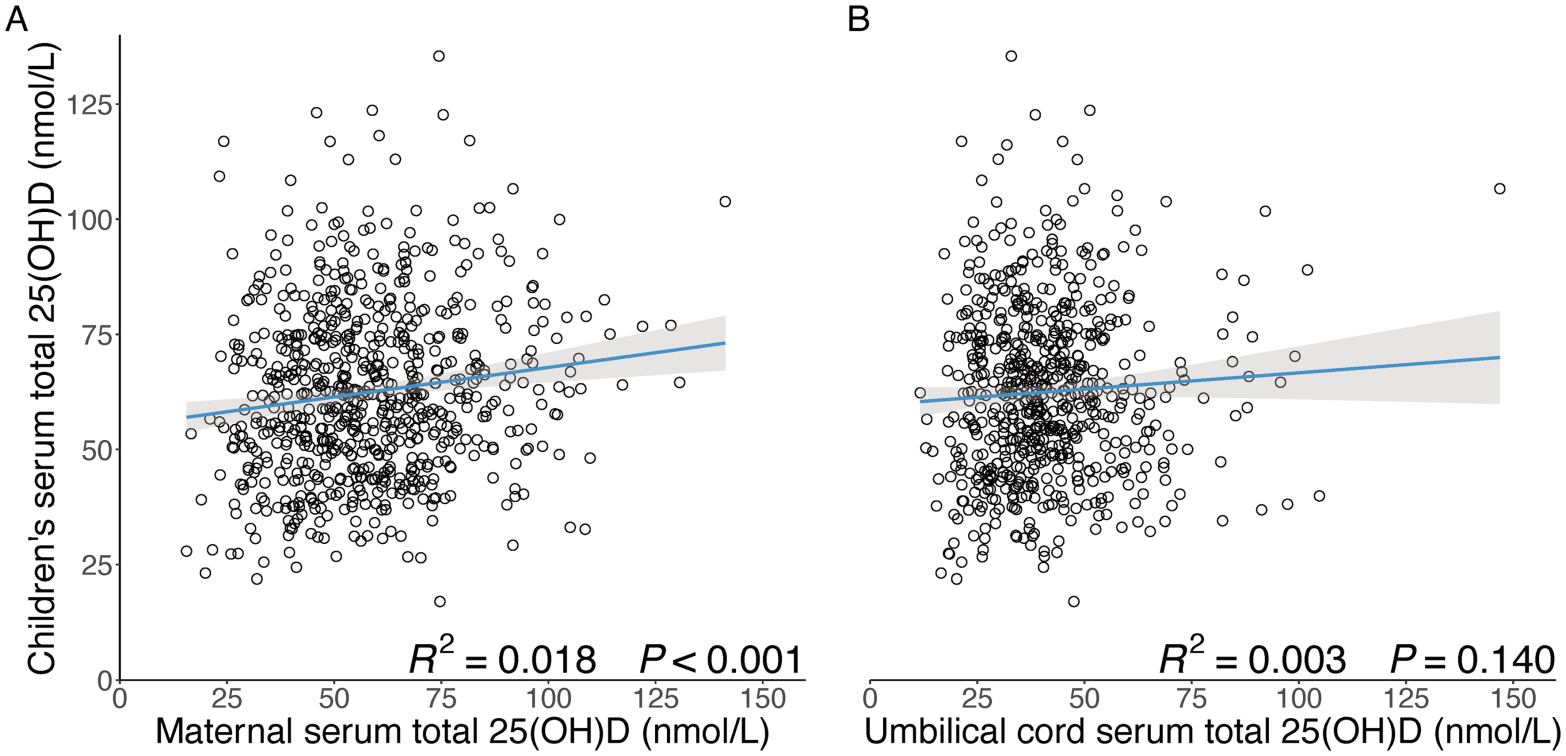
Associations of maternal and umbilical cord serum total 25(OH)D levels with children’s serum total 25(OH)D levels at an average of 7 years. **(A)** Linear correlation between maternal serum total 25(OH)D level with children’s serum total 25(OH)D level at an average of 7 years; (**B)** Linear correlation between umbilical cord serum total 25(OH)D level with children’s serum total 25(OH)D level at an average of 7 years. Grey areas represent the 95% CI of linear models. *p*-value for fitted linear models, and *p* < 0.05 was considered significant.

As shown in Table 1, none of the cardiometabolic risk factors in early childhood were associated with maternal serum total 25(OH)D levels in mid-gestation. However, there was a trend for an increase in children’s vitamin D with increasing maternal serum total 25(OH)D levels in mid-gestation, showing a weak linear correlation (R^2^ = 0.018, *p* < 0.001) (Figure 1A), supporting the possibility that the children’s vitamin D concentration may act as a mediator lie on the pathway between maternal serum vitamin D concentration and childhood cardiometabolic risks.

**Table 1:** Children’s characteristics at 7 years of age by maternal serum total 25(OH) level during pregnancy.

Variable	Maternal serum total 25(OH)D level (nmol/L)				*p*
	<25 (*n* = 16)	25- < 50 (*n* = 328)	50- < 75 (*n* = 397)	≥75 (*n* = 156)	
Age at follow-up (year)	7.03 ± 0.36	6.96 ± 0.40	6.96 ± 0.43	6.96 ± 0.49	0.852
Boy (%)	9 (56.25%)	163 (49.70%)	190 (47.86%)	75 (48.08%)	0.889
Anthropometry					
Height (cm)	125.57 ± 3.97	124.29 ± 4.76	124.10 ± 5.16	123.81 ± 5.37	0.612
BMI (kg/m^2^)	15.20 ± 2.52	15.11 ± 2.44	14.99 ± 2.21	15.11 ± 2.35	0.984
Waist-hip ratio	0.85 ± 0.04	0.84 ± 0.05	0.84 ± 0.04	0.84 ± 0.04	0.586
Body fat%	20.02 ± 10.27	19.07 ± 7.18	18.93 ± 6.97	18.98 ± 6.80	0.998
Sum of skinfold thickness (mm)	37.79 ± 23.95	36.42 ± 17.82	35.76 ± 16.81	36.31 ± 16.94	0.995
Blood pressure & arterial stiffness					
Systolic BP (mmHg)	102.33 ± 9.65	101.79 ± 9.27	102.15 ± 8.90	101.53 ± 8.25	0.934
Diastolic BP (mmHg)	60.71 ± 5.93	61.55 ± 8.66	62.07 ± 7.55	62.40 ± 7.77	0.298
Prehypertension /hypertension (%) ^*^	1 (6.25%)	57 (17.54%)	55 (13.89%)	21 (13.82%)	0.445 ^a^
Carotid-femoral PWV (m/s)	4.58 ± 0.59	4.72 ± 0.62	4.77 ± 0.61	4.78 ± 0.59	0.334
Children’s glycemia status at OGTT					
Fasting PG level (mmol/L)	4.62 ± 0.31	4.56 ± 0.37	4.58 ± 0.33	4.57 ± 0.37	0.743
15-min PG level (mmol/L)	6.68 ± 0.95	7.08 ± 1.24	7.04 ± 1.18	7.08 ± 1.17	0.646
30-min PG level (mmol/L)	6.73 ± 1.31	7.52 ± 1.50	7.68 ± 1.47	7.60 ± 1.61	0.082 ^b^
1-h PG level (mmol/L)	5.46 ± 1.22	5.86 ± 1.43	5.99 ± 1.57	5.90 ± 1.67	0.457
2-h PG level (mmol/L)	5.25 ± 0.84	5.27 ± 0.98	5.31 ± 0.97	5.28 ± 0.94	0.815
Abnormal glucose tolerance (%) ^#^	0 (0.00%)	8 (2.62%)	5 (1.33%)	3 (2.10%)	0.568 ^a^
Matsuda ISI	20.08 ± 13.28	16.08 ± 9.18	15.76 ± 8.37	16.50 ± 9.71	0.670
HOMA-IR at OGTT	0.67 ± 0.33	0.84 ± 0.83	0.79 ± 0.73	0.80 ± 0.70	0.900
HOMA-*β* at OGTT	58.52 ± 27.95	82.03 ± 83.23	75.04 ± 66.88	76.78 ± 63.19	0.782
IGI at 30-min	75.71 ± 40.53	83.85 ± 91.98	74.79 ± 90.72	75.82 ± 81.91	0.180
Lipid profile (mmol/L)					
TC	4.38 ± 0.61	4.50 ± 0.75	4.48 ± 0.74	4.42 ± 0.72	0.522
TG	0.78 ± 0.28	0.74 ± 0.34	0.74 ± 0.34	0.76 ± 0.33	0.795
HDL	1.53 ± 0.28	1.66 ± 0.36	1.67 ± 0.34	1.65 ± 0.35	0.374
LDL	2.48 ± 0.50	2.51 ± 0.65	2.48 ± 0.67	2.43 ± 0.62	0.520
Ambient solar radiation at blood taken and children’s vitamin D level					
Ambient solar radiation (MJ/m^2^) ^$^	338.67 ± 45.56	381.60 ± 88.05	390.79 ± 71.63	410.62 ± 77.17	<0.001
Serum total 25(OH)D	56.67 ± 29.88	60.08 ± 18.28	62.72 ± 18.45	67.32 ± 18.26	0.003

Continuous and categorical variables were expressed as mean ± SD and n (proportion in %), respectively.

Continuous variables were compared by the Kruskal-Wallis test unless otherwise indicated, and categorical variables were compared using the chi-square test unless otherwise indicated.

^*^Prehypertension/hypertension is defined as children’s blood pressure percentile ≥ 90th percentile.

^#^Abnormal glucose tolerance includes impaired fasting glucose (FPG between 5.6 and <7.0 mmol/L), impaired glucose tolerance (2 h PG between 7.8 and 11.0 mmol/L), and diabetes (FPG ≥ 7.0 mmol/L or 2 h PG ≥ 11.0 mmol/L).

^$^Monthly global solar radiation recorded at the Hong Kong Observatory at the time of the oral glucose tolerance test during the study period was obtained from the public domain.

^a^Fisher’s exact test was utilized for group comparison.

^b^ANOVA test was utilized for group comparison.

25(OH)D, 25-hydroxyvitamin D; BMI, body mass index; BP, blood pressure; PWV, pulse wave velocity; PG, plasma glucose; ISI, insulin sensitivity index; HOMA-IR, Homeostatic Model Assessment for Insulin Resistance; HOMA-β, Homeostatic Model Assessment for β-cell function; IGI, insulinogenic index; TC, total cholesterol; TG, triglyceride; HDL, high-density lipoprotein; LDL, low-density lipoprotein.

**Figure 2:**
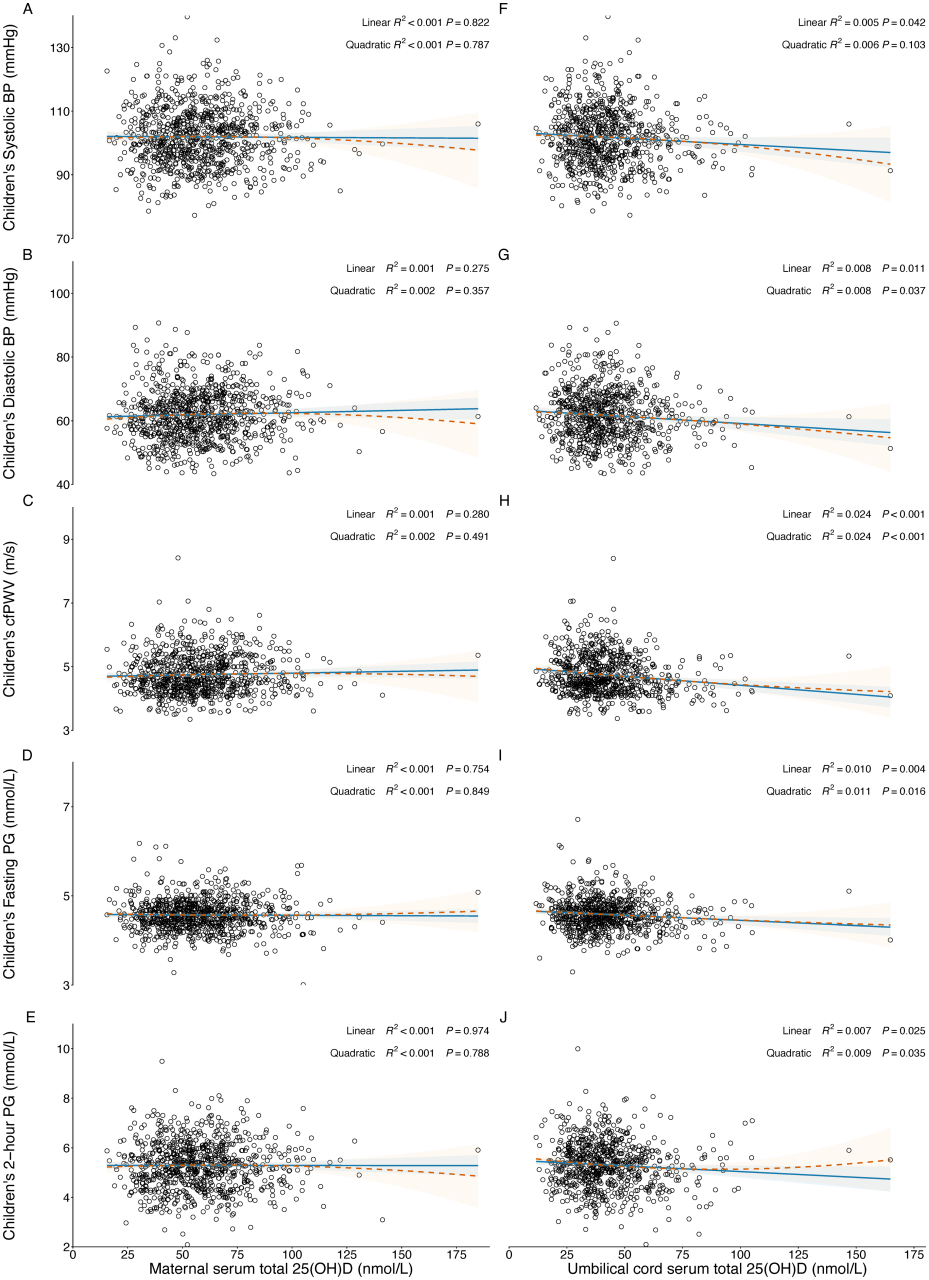
Linear and quadratic associations between in-utero serum total 25(OH)D levels and children’s cardiometabolic risks at around 7 years of age. The solid line represents a linear association, and the dashed line represents a quadratic association. Blue and orange areas represent the 95% CI of linear models and quadratic models, respectively. **(A–E)** Associations of maternal serum total 25(OH)D concentration with children’s systolic BP, diastolic BP, cfPWV, fasting PG, and 2-h PG at the time of oral glucose tolerance test; **(F–J)** associations of umbilical cord serum total 25(OH)D concentration with children’s systolic BP, diastolic BP, cfPWV, fasting PG, and 2-h PG at the time of oral glucose tolerance test. PG, plasma glucose; BP, blood pressure; cfPWV, carotid-femoral pulse wave velocity.

Figures 2A–E depicts both the linear and quadratic associations of maternal serum 25(OH)D concentration measured at 24–32 weeks of gestation with children’s BP, cfPWV, and glucose levels. However, there were no significant linear or quadratic associations between maternal serum 25(OH)D level measured at 24–32 gestational weeks and children’s systolic BP (Linear: R^2^ < 0.001, *p* = 0.822; Quadratic: R^2^ < 0.001, *p* = 0.787), diastolic BP (Linear: R^2^ = 0.001, *p* = 0.275; Quadratic: R^2^ = 0.002, *p* = 0.357), cfPWV (Linear: R^2^ = 0.001, *p* = 0.280; Quadratic: R^2^ = 0.001, *p* = 0.491), fasting PG (Linear: R^2^ < 0.001, *p* = 0.754; Quadratic: R^2^ < 0.001, *p* = 0.849), and 2-h PG (Linear: R^2^ < 0.001, *p* = 0.974; Quadratic: R^2^ < 0.001, *p* = 0.788) at around 7 years of age.

### Distribution of children’s cardiometabolic risk factors by umbilical cord serum vitamin D levels at birth

In contrast to maternal vitamin D levels in mid-gestation, umbilical cord serum 25(OH)D was not associated with children’s vitamin D levels (R^2^ = 0.003, *p* = 0.140) (Figure 1B), which did not support treating children’s vitamin D concentration as a mediator in the analysis between umbilical cord vitamin D concentration and childhood cardiometabolic risks. Thus, children’s vitamin D concentration at around age 7 was retained as an adjusted covariate in the subsequent analysis. Moreover, as detailed in Table 2, significant differences across categories of umbilical cord serum total 25(OH)D concentration were observed for children’s cfPWV, as well as the fasting PG and 2-h PG levels at OGTT among groups. In addition, there were trends for an increase in children’s systolic BP, diastolic BP, cfPWV, as well as the fasting PG and 2-h PG levels at OGTT, with decreasing umbilical cord serum total 25(OH)D levels (*P* for trend < 0.05).

**Table 2:** Children’s characteristics at 7 years of age by umbilical cord serum total 25(OH) level.

Variable	Umbilical cord serum total 25(OH)D level (mmol/L)				*p*
	<25 (*n* = 79)	25– < 50 (*n* = 520)	50– < 75 (n = 171)	≥75 (*n* = 33)	
Age at follow-up (year)	6.97 ± 0.45	6.99 ± 0.45	6.94 ± 0.40	6.87 ± 0.27	0.100
Boy (%)	40 (50.63%)	265 (50.96%)	98 (57.31%)	14 (42.42%)	0.331
Anthropometry					
Height (cm)	125.45 ± 4.49	124.24 ± 5.07	123.98 ± 4.86	123.37 ± 5.24	0.167
BMI (kg/m^2^)	14.98 ± 2.02	15.10 ± 2.36	15.06 ± 2.16	14.70 ± 1.72	0.921
Waist-hip ratio	0.83 ± 0.06	0.84 ± 0.04	0.84 ± 0.04	0.84 ± 0.04	0.912
Body fat%	18.55 ± 5.76	19.16 ± 7.29	18.41 ± 6.37	17.73 ± 4.92	0.754
Sum of skinfold thickness (mm)	35.34 ± 13.88	36.61 ± 17.97	34.45 ± 15.32	32.49 ± 11.51	0.468
Blood pressure & arterial stiffness					
Systolic BP (mmHg)	102.35 ± 8.76	102.21 ± 9.14	100.67 ± 8.75	99.28 ± 6.90	0.071
Diastolic BP (mmHg)	62.89 ± 7.81	62.01 ± 8.27	60.60 ± 7.71	59.37 ± 5.37	0.057
Prehypertension /hypertension (%) ^*^	16 (20.51%)	78 (15.18%)	18 (10.59%)	2 (6.06%)	0.096 ^a^
Carotid-femoral PWV (m/s)	4.86 ± 0.54	4.80 ± 0.64	4.59 ± 0.51	4.43 ± 0.51	<0.001
Children’s glycemia status at OGTT					
Fasting PG level (mmol/L)	4.67 ± 0.36	4.59 ± 0.39	4.55 ± 0.34	4.45 ± 0.34	0.045
15-min PG level (mmol/L)	7.14 ± 1.13	7.05 ± 1.18	6.89 ± 1.09	6.78 ± 1.24	0.231
30-min PG level (mmol/L)	7.52 ± 1.39	7.63 ± 1.51	7.49 ± 1.52	7.61 ± 1.48	0.740
1-h PG level (mmol/L)	5.90 ± 1.35	6.04 ± 1.49	5.92 ± 1.62	5.75 ± 1.84	0.533
2-h PG level (mmol/L)	5.33 ± 0.94	5.37 ± 0.95	5.16 ± 0.93	5.04 ± 1.08	0.031
Abnormal glucose tolerance (%) ^#^	2 (2.63%)	9 (1.86%)	2 (1.22%)	0 (0.00%)	0.817 ^a^
Matsuda ISI	16.62 ± 9.43	15.61 ± 9.14	16.05 ± 8.32	18.19 ± 9.70	0.278
HOMA-IR at OGTT	0.77 ± 0.68	0.83 ± 0.74	0.84 ± 1.00	0.64 ± 0.36	0.479
HOMA-β at OGTT	66.17 ± 55.59	78.41 ± 68.00	81.64 ± 93.83	80.69 ± 64.95	0.149
IGI at 30-min	83.93 ± 82.33	77.09 ± 91.05	75.26 ± 72.95	99.06 ± 162.52	0.599
Lipid profile (mmol/L)					
TC	4.51 ± 0.69	4.48 ± 0.73	4.39 ± 0.64	4.59 ± 0.79	0.384
TG	0.79 ± 0.29	0.74 ± 0.35	0.72 ± 0.27	0.69 ± 0.38	0.088
HDL	1.62 ± 0.33	1.67 ± 0.36	1.65 ± 0.33	1.74 ± 0.31	0.255
LDL	2.53 ± 0.63	2.48 ± 0.63	2.42 ± 0.54	2.54 ± 0.68	0.579
Ambient solar radiation at blood taken and children’s vitamin D level					
Ambient Solar radiation (MJ/m^2^) ^$^	368.94 ± 69.12	378.49 ± 75.11	400.97 ± 81.17	403.16 ± 78.91	0.001
Serum total 25(OH)D (nmol/L)	58.95 ± 20.32	62.84 ± 18.21	62.45 ± 18.83	67.22 ± 21.13	0.287

Continuous and categorical variables were expressed as mean ± SD and n (proportion in %), respectively.

Continuous variables were compared by the Kruskal-Wallis test unless otherwise indicated, and categorical variables were compared using the chi-square test unless otherwise indicated.

^*^Prehypertension/hypertension is defined as children’s blood pressure percentile ≥ 90th percentile.

^#^Abnormal glucose tolerance includes impaired fasting glucose (FPG between 5.6 and <7.0 mmol/L), impaired glucose tolerance (2 h PG between 7.8 and 11.0 mmol/L), and diabetes (FPG ≥ 7.0 mmol/L or 2 h PG ≥ 11.0 mmol/L).

^$^Monthly global solar radiation recorded at the Hong Kong Observatory at birth during the study period was obtained from the public domain.

^a^Fisher’s exact test was utilized for group comparison.

25(OH)D, 25-hydroxyvitamin D; BMI, body mass index; BP, blood pressure; PWV, pulse wave velocity; PG, plasma glucose; ISI, insulin sensitivity index; HOMA-IR, Homeostatic Model Assessment for Insulin Resistance; HOMA-β, Homeostatic Model Assessment for β-cell function; IGI, insulinogenic index; TC, total cholesterol; TG, triglyceride; HDL, high-density lipoprotein; LDL, low-density lipoprotein.

Figures 2F–J depicts the linear and quadratic associations of umbilical cord serum 25(OH)D levels with children’s BP, cfPWV, fasting PG, and 2-h PG at around 7 years of age. Significant negative linear associations were observed between umbilical cord serum total 25(OH)D levels and children’s systolic BP (R^2^ = 0.005, *p* = 0.042), diastolic BP (R^2^ = 0.008, *p* = 0.011), cfPWV (R^2^ = 0.024, *p* < 0.001), fasting PG (R^2^ = 0.010, *p* = 0.004), and 2-h PG (R^2^ = 0.007, *p* = 0.025) at around 7 years of age. Quadratic associations were also significant for children’s diastolic BP (R^2^ = 0.008, *p* = 0.037), cfPWV (R^2^ = 0.024, *p* < 0.001), fasting PG (R^2^ = 0.011, *p* = 0.016), and 2-h PG (R^2^ = 0.009, *p* = 0.035), but not for systolic BP (R^2^ = 0.006, *p* = 0.103). However, the quadratic models did not provide a better fit than the linear models, as the R^2^ values of the quadratic models were not significantly greater than those of the linear models (diastolic BP: 0.008 vs. 0.008, *p* = 0.744; cfPW: 0.024 vs. 0.024, *p* = 0.654; fasting PG: 0.011 vs. 0.010, *p* = 0.841; 2-h PG: 0.009 vs. 0.007, *p* = 0.196), indicating no substantial improvement in model fit with the quadratic specification.

### Associations between in-utero serum total 25(OH)D levels and children’s cardiometabolic risks at around 7 years of age

Table 3 shows the association between maternal and umbilical cord serum 25(OH)D levels and children’s cardiometabolic risks by linear regression models. No significant associations were observed between maternal serum 25(OH)D in mid-gestation and any children’s cardiometabolic risk factors. However, inverse associations were observed between umbilical cord serum 25(OH)D levels and childhood cardiometabolic risks that every 1-SD (18.0 nmol/L) increase of umbilical cord serum total 25(OH)D level was associated with a reduction in children’s diastolic BP, cfPWV, and fasting PG by 0.73 mmHg (95% CI [−1.44, −0.02], *p* = 0.044), 0.08 m/s (95% CI [−0.14, −0.03], *p* = 0.004), and 0.04 mmol/L (95% CI [−0.07, −0.01], *p* = 0.017), respectively, after covariate adjustment, including ambient solar radiation levels at birth and children’s vitamin D status at around age 7. However, after FDR correction, only the associations with cfPWV and fasting PG remained significant. Additionally, associations with systolic BP and 2-h PG were not significant after multivariable adjustment.

**Table 3:** Associations of maternal and umbilical cord serum total 25(OH)D levels with children’s cardiometabolic risks at 7 years of age.

Outcome	Maternal serum total 25(OH)D				Umbilical cord serum total 25(OH)D			
	Univariate		Multivariate		Univariate		Multivariate	
	β^a^ (95% CI)	*p*	β^a^ (95% CI)	*p*	β^b^ (95% CI)	*p*	β^b^ (95% CI)	*p*
BP & arterial stiffness								
Systolic BP (mmHg) ^#^	−0.07 (−0.66, 0.52)	0.822	−0.07 (−0.65, 0.51)	0.814	−0.70 (−1.38, −0.02)	0.042	−0.43 (−1.21, 0.34)	0.273
Diastolic BP (mmHg) ^#^	0.29 (−0.23, 0.82)	0.275	0.42 (−0.12, 0.95)	0.125	−0.79 (−1.40, −0.18)	0.011	−0.73 (−1.44, −0.02)	0.044
Carotid-femoral PWV (m/s) ^&^	0.02 (−0.02, 0.06)	0.280	0.02 (−0.03, 0.06)	0.451	−0.10 (−0.15, −0.06)	<0.001	−0.08 (−0.14, −0.03)	0.004 ^c^
Glucose (mmol/L)^*^								
Fasting PG	0.00 (−0.03, 0.02)	0.754	0.00 (−0.03, 0.02)	0.796	−0.04 (−0.07, −0.01)	0.004	−0.04 (−0.07, −0.01)	0.017 ^c^
2-h PG	0.00 (−0.07, 0.06)	0.974	−0.02 (−0.08, 0.05)	0.658	−0.08 (−0.16, −0.01)	0.025	−0.09 (−0.18, 0.00)	0.056

^a^β corresponded to an increase of 1 SD in maternal serum 25(OH)D level (19.9 nmol/L).

^b^β corresponded to an increase of 1 SD in umbilical cord serum 25(OH)D level (18.0 nmol/L).

^c^*p*-value remains < 0.05 after using the Benjamini-Hochberg correction with FDR = 0.05.

BP, blood pressure; PG, plasma glucose; PWV, pulse wave velocity; FDR, false discovery rate.

^#^Adjusted for maternal age at pregnancy, gestational weight gain z-score, hypertensive disorders in pregnancy, mean solar radiation at blood taken, breastfeeding, as well as children’s sex, age, exercise level, height, and serum total 25(OH)D at 7 years of age (not adjusted for maternal serum total 25(OH)D).

^&^Adjusted for maternal age at pregnancy, gestational weight gain z-score, hypertensive disorders in pregnancy, mean solar radiation at blood taken, breastfeeding, as well as children’s sex, age, exercise level, height, serum total 25(OH)D at 7 years of age (not adjusted for maternal serum total 25(OH)D), and dyslipidaemia.

^*^Adjusted for maternal age at pregnancy, gestational weight gain z-score, sum of glucose z-score at OGTT at pregnancy, mean solar radiation at blood taken, breastfeeding, as well as children’s sex, age, exercise level, BMI, and serum total 25(OH)D at 7 years of age (not adjusted for maternal serum total 25(OH)D).

## Discussion

In the present study of Chinese mother–child pairs, we observed that lower umbilical cord serum total 25(OH)D levels were associated with higher diastolic BP, cfPWV, and fasting PG in children at around 7 years of age, independent of ambient solar radiation at birth and children’s vitamin D status at around age 7. However, only the associations with cfPWV and fasting PG remained significant after FDR correction. In contrast, maternal serum vitamin D concentration at 24–32 weeks of gestation was not associated with any childhood cardiometabolic risk factors. Our findings indicated that hypovitaminosis D in umbilical cord serum, but not in maternal serum at 24–32 weeks of gestation, might contribute to childhood vascular dysfunction and glucose dysregulation at around age 7.

Vitamin D plays an important role in regulating BP and arterial stiffness. Evidence from animal and cellular studies suggests that vitamin D might influence BP by modulating the renin-angiotensin-aldosterone system, regulating the proliferation of vascular smooth muscle cells and cardiomyocytes, and suppressing the release of parathyroid hormone (38–42). Moreover, parental vitamin D deficiency during pregnancy in rats has been shown to increase offspring BP through hypermethylation of the promoter region of the Pannexin-1 gene, impairing endothelial relaxation. Regarding arterial stiffness, *in vitro* and animal studies also indicated that vitamin D might modulate endothelial cell function by reducing the expression of adhesion molecules, providing protection against advanced glycation products, reducing endothelium-dependent contractions, and regulating calcium influx (43–45), thereby potentially influencing the development of arterial stiffness. These mechanisms provide biological plausibility for a role of early-life vitamin D exposure in later vascular health. Since fetal 25(OH)D levels are primarily dependent on maternal vitamin D status (12), it is postulated that intrauterine hypovitaminosis D might have lasting adverse effects on offspring’s cardiovascular risk in later life.

An inverse association between maternal vitamin D levels during pregnancy and children’s BP has been found in three large cohorts that utilized liquid chromatography–tandem mass spectrometry (15, 18, 46), methods considered more accurate than immunoassays, but not in other observational studies (14, 17, 19, 20, 47, 48). Of note, in the large observational Amsterdam study by Hrudey et al. (48) and the Dutch study by Miliku et al. (17), no association was observed between maternal vitamin D in pregnancy and children’s BP at 5–6 years. It may be due to the adjustment for multivitamin supplementation in pregnancy, which may mask the true association. Apart from multivitamin supplementation, studies with small sample sizes, including our study, may also limit the ability to detect associations (14, 19, 20, 47). Further investigations with large prospective cohort studies are needed to clarify the association between maternal vitamin D concentrations during pregnancy and children’s BP.

Regarding umbilical cord vitamin D, most of the observational studies have reported an inverse association with children’s BP (16, 46, 49), while only a few have not (18). Notably, Larsen et al. observed an inverse association between umbilical cord vitamin D and children’s BP among girls at age 3 (46), whereas Pedersen et al., also in the Odense Child cohort, did not observe such at age 5, even in girls (18). This inconsistency may be potentially due to a lack of adjustment for season of birth and children’s age at BP measurement in Pedersen et al.’s study (18). Since umbilical cord vitamin D varies across seasons (27), failing to adjust for season at birth may bias the estimation of the effects of ambient solar radiation levels on the results. In our study, the association with diastolic BP was modest and did not persist after correction for multiple testing. Although this pattern broadly aligns with previous studies, the magnitude of the association varied across studies. Specifically, we observed a 0.73 mmHg increase in diastolic BP at around age 7 with every 18-nmol/L decrease in umbilical cord vitamin D, whereas Odense Child Cohort study reported only a 0.4 mmHg increase in diastolic BP at age 3 with per 10-nmol/L decrease in umbilical cord vitamin D (46), and the Healthy Start study showed a 1.7 mmHg increase in diastolic BP at age 5 with per 25-nmol/L decrease in umbilical cord vitamin D (16). These differences may, at least in part, be attributed to the age at BP assessment. Collectively, our findings provide a potential association between umbilical cord 25(OH)D levels and children’s BP, particularly diastolic BP. However, this interpretation should be made cautiously, and further investigations are warranted, given the absence of a significant association with systolic BP after covariate adjustment, no significant difference in prevalence of prehypertension/hypertension, and the attenuation of the diastolic BP association after FDR correction.

With respect to children’s arterial stiffness, we did not observe any association of maternal vitamin D levels in pregnancy with childhood arterial stiffness, in line with findings from studies conducted in the UK and Spain (19, 47). In contrast, we found an inverse association between umbilical cord serum total 25(OH)D levels and children’s cfPWV at around age 7, independent of ambient solar radiation at birth, children’s BMI, exercise, and children’s vitamin D status. Notably, this finding differs from the Healthy Start study by Sauder et al., which did not observe an association between umbilical cord vitamin D levels and children’s cfPWV at around age 5, potentially due to the younger age of participants and a smaller sample size (16). Although our findings indicated a potential effect of umbilical cord vitamin D levels on children’s arterial stiffness at around age 7 in a relatively large cohort study, the effect size was small in our study, a 0.08 m/s increase with every 18-nmol/L decrease in umbilical cord serum total 25(OH)D. Thus, further investigation is needed to determine the consistency of this association and to clarify the causal relationship.

Beyond cardiovascular risk factors, animal studies indicated that vitamin D exclusively affected the insulin response to glucose stimulation with little or no impact on basal insulinemia (50, 51). Moreover, maternal vitamin D deficiency in mice suppressed fetal hepatic AMPK/KET signaling axis by reducing superoxide dismutase 3 levels released from the placenta, potentially elevating offspring glucose levels (52). These findings suggest that in utero vitamin D levels might be associated with offspring glucose levels later in life. However, the results were not supported by human studies (14, 15, 48), and our results also do not support an association between maternal vitamin D levels in mid-gestation and children’s glucose levels at OGTT. Conversely, we found an inverse association between umbilical cord serum total 25(OH)D levels and children’s fasting PG levels at around age 7, regardless of children’s vitamin D levels. Although our study is the first study to show the long-term effects of umbilical cord vitamin D levels on children’s fasting PG, the observed effect size was still small, a 0.04 mmol/L increase for every 18-nmol/L decrease in umbilical cord vitamin D, and the association with 2-h PG at OGTT was no longer significant after adjustment. In addition, the prevalence of abnormal glucose tolerance, insulin sensitivity, insulin resistance, *β*-cell function, or the insulinogenic index did not differ significantly among umbilical cord vitamin D groups. Therefore, the impact of umbilical cord vitamin D levels, rather than maternal serum levels, on children’s fasting PG levels and other glucose metabolism outcomes warrants confirmation in further studies.

To contextualize the clinical relevance of our findings, it is crucial to recognize that cardiometabolic risk factors identified in early childhood may extend into adulthood and have been associated with later cardiovascular and metabolic diseases. Extensive evidence demonstrated that elevated childhood BP and increased arterial stiffness are significantly associated with a higher risk of adult hypertension, atherosclerosis, and premature cardiovascular events in later life (21, 22). Similarly, fasting PG during childhood, even within the normoglycemic range, has been identified as an independent predictor for incident type 2 diabetes mellitus in adulthood (23). Therefore, the adverse shifts in diastolic BP, cfPWV, and fasting PG associated with lower in utero vitamin D levels in our study might suggest early subclinical variation in childhood cardiometabolic health, though they should be interpreted cautiously, given the small magnitude. In addition, with significant associations only observed in a subset of the cardiometabolic risk factors, our findings should not be interpreted as sufficient evidence to support routine screening of umbilical cord vitamin D levels for predicting future cardiometabolic risks. Rather, umbilical cord vitamin D may warrant further investigation as a potential marker of early-life exposure, and its possible role in the identification of higher-risk children should be evaluated in future studies with repeated measurements and longer follow-up. Currently, these findings do not support specific recommendations regarding pediatric monitoring or preventive interventions, which should await further evidence and confirmation.

### Strengths and limitations

This mother–child follow-up cohort was prospectively designed with comprehensive clinical assessments of childhood cardiometabolic risk factors. Moreover, LC–MS/MS was used for maternal, umbilical cord, and children’s vitamin D measurement in our study, ensuring sensitivity, accuracy, and reliability. Furthermore, the detailed measurements collected in early life and childhood, as well as exercise level and ambient solar radiation levels, both highly related to vitamin D metabolism, allowed meaningful adjustment of confounders, which provided objective evidence to our association study.

However, this study has some limitations. First, over one-third (38.9%) of the participants in the original HAPO Study lost follow-up due to being non-eligible, declining the follow-up visit, or being uncontactable. Although baseline comparisons revealed statistically significant differences between the retained participants and those lost to follow-up in certain maternal characteristics, such as age, prepregnant BMI, 2-h PG, and total 25(OH)D levels at OGTT, the standardized mean differences (SMDs) for all evaluated variables were small (SMDs < 0.25, Supplementary Table 1), indicating that these statistical differences might not be clinically meaningful. Notably, no significant differences were observed in our primary exposure of interest, umbilical cord 25(OH)D levels at birth (*p* = 0.236, SMD = 0.0654), or in other important neonatal characteristics. Nevertheless, this substantial attrition might still affect the internal validity of our findings; thus, the results should be interpreted with caution. Second, we only included Chinese participants in our study, which limited the generalizability of our findings to other populations. Third, the lack of records of dietary vitamin D supplementation may introduce bias in the associations, although this is not common in our population (53). Moreover, as maternal vitamin D levels were measured only once in mid-gestation of pregnancy in our study, we were unable to evaluate the cumulative effects of vitamin D throughout pregnancy on the offspring cardiometabolic risk factors, nor to identify potential overall and critical windows during which intrauterine hypovitaminosis D influences these factors. In addition, although we classified maternal and umbilical cord serum total 25(OH) using the same guideline-based thresholds, these cutoffs have not been universally accepted for umbilical cord serum vitamin D. Thus, the categories of umbilical cord vitamin D should be interpreted as standardized exposure strata rather than definitive clinical diagnostic thresholds. Finally, the interval between birth and the follow-up assessment at around 7 years of age is a considerable gap period. The lack of continuous assessments of childhood nutrition, detailed growth and development measurements, a history of infections, and other unmeasured potential risk factors during this long period might influence both childhood cardiometabolic risk and vitamin D levels, increasing the risk of residual bias. Thus, future studies should incorporate more detailed longitudinal follow-up from early to later periods to better elucidate these associations and confirm our findings.

## Conclusion

Given that inverse associations were only observed between umbilical cord vitamin D and children’s cardiometabolic risk factors, our findings suggest that umbilical cord vitamin D levels at birth might serve as a more proximate marker of early-life exposure relevant to childhood vascular function and glucose metabolism than maternal vitamin D levels measured at a single time point during pregnancy. This may raise the possibility that umbilical cord vitamin D might capture fetal vitamin D exposure more comprehensively than a single maternal measurement. However, further longitudinal studies with repeated maternal vitamin D measurements across pregnancy are needed to clarify whether cumulative or time-specific in utero vitamin D exposure influences offspring vascular function and glucose metabolism. In addition, further research is required to determine whether umbilical cord vitamin D has potential value as an early biomarker for identifying children at higher cardiometabolic risk.

## Data Availability

The datasets presented in this article are not readily available because restrictions apply to the availability of some, or all data generated or analyzed during this study to preserve patient confidentiality or because they were used under license. The corresponding authors will, on request, detail the restrictions and any conditions under which access to some data may be provided. Requests to access the datasets should be directed to Chi-Chiu Wang, ccwang@cuhk.edu.hk.
