# Supplementary material for: Associations of umbilical cord vitamin D levels with childhood cardiometabolic risks: a longitudinal mother–child study
Source: Front Nutr. 2026 Apr 13;13:1777909. doi: 10.3389/fnut.2026.1777909 (PMC13111193; doi:10.3389/fnut.2026.1777909)
Supplement: Supplementary file 1 [file Table_1.docx]

# Supplements

## Supplementary Table 1. Comparison of baseline characteristics between participants of the HAPO follow-up and those lost to follow-up at 7 years postpartum

|  | **Lost follow-up at 7 years postpartum** | **Follow-up at 7 years postpartum** | **P-value** | **SMD** |
| --- | --- | --- | --- | --- |
| Sample size | 637 | 973 |  |  |
| Maternal age (year) | 30.25 ± 5.11 | 31.30 ± 4.64 | <0.001 | 0.2153 |
| Prepregnant BMI (kg/m^2^) | 20.33 ± 2.79 | 20.88 ± 2.85 | <0.001 | 0.1940 |
| Gestational weight gain (kg) | 15.71 ± 4.82 | 15.12 ± 4.36 | 0.012 | 0.1286 |
| Fasting PG (mmol/L) | 4.35 ± 0.32 | 4.36 ± 0.33 | 0.269 | 0.0567 |
| 1-hour PG (mmol/L) | 7.51 ± 1.70 | 7.68 ± 1.65 | 0.050 | 0.0996 |
| 2-hour PG (mmol/L) | 6.36 ± 1.21 | 6.65 ± 1.32 | <0.001 | 0.2306 |
| Sum of glucose z-score | -0.23 ± 2.27 | 0.15 ± 2.41 | 0.002 | 0.1636 |
| Gestational diabetes mellitus, n (%) | 71 (11.15%) | 143 (14.70%) | 0.048 | 0.1060 |
| Hypertensive disorders in pregnancy, n (%) | 39 (6.12%) | 81 (8.32%) | 0.122 | 0.0851 |
| Maternal serum 25(OH)D at OGTT (nmol/L) | 54.96 ± 18.93 | 57.80 ± 19.88 | 0.006 | 0.1460 |
| Ambient Solar radiation at OGTT (MJ/m^2^) | 391.16 ± 75.99 | 388.66 ± 79.85 | 0.532 | 0.0320 |
| Gestational age (week) | 39.36 ± 1.52 | 39.36 ± 1.50 | 0.931 | 0.0044 |
| Birth weight (g) | 3,142.51 ± 419.55 | 3,180.04 ± 435.09 | 0.086 | 0.0878 |
| Boys, n (%) | 302 (47.41%) | 469 (48.20%) | 0.795 | 0.0158 |
| Umbilical serum 25(OH)D at delivery(nmol/L) | 43.65 ± 20.12 | 42.44 ± 16.51 | 0.236 | 0.0654 |
| Ambient Solar radiation at delivery (MJ/m^2^) | 384.70 ± 73.87 | 382.98 ± 77.22 | 0.656 | 0.0228 |

Abbreviation: 25(OH)D, 25-hydroxyvitamin D; BMI, body mass index; PG, plasma glucose; OGTT, oral glucose tolerance test; SMD, standardized mean difference.

Continuous variables are expressed as mean ± SD and categorical variables as n (%).

Comparisons between groups were performed using Student’s t-test for continuous variables and Chi-square tests for categorical variables.

P < 0.05 was considered statistically significant.

## Supplementary Table 2. Maternal, neonatal, and children’s baseline characteristics.

|  | **Overall** |
| --- | --- |
| Sample size | 973 |
| **Maternal characteristics in the original HAPO study** |  |
| Age (year) | 31.30 ± 4.64 |
| Prepregnant BMI (kg/m^2^) | 20.88 ± 2.85 |
| Gestational weight gain (kg) | 15.12 ± 4.36 |
| Fasting PG (mmol/L) | 4.36 ± 0.33 |
| 1-hour PG (mmol/L) | 7.68 ± 1.65 |
| 2-hour PG (mmol/L) | 6.65 ± 1.32 |
| Sum of glucose z-score | 0.15 ± 2.41 |
| Maternal serum total 25(OH)D (nmol/L) | 57.80 ± 19.88 |
| Ambient solar radiation at OGTT (MJ/m^2^) ^$^ | 388.66 ± 79.85 |
| Gestational diabetes mellitus, n (%) | 143 (14.70%) |
| Hypertensive disorders in pregnancy, n (%) | 81 (8.32%) |
| Gestational age (week) | 39.36 ± 1.50 |
| **Neonatal characteristics at birth** |  |
| Birth weight (g) | 3,180.04 ± 435.09 |
| Boys, n (%) | 469 (48.20%) |
| Umbilical cord serum total 25(OH)D (nmol/L) | 42.44 ± 16.51 |
| Ambient solar radiation at delivery (MJ/m^2^) ^$^ | 382.98 ± 77.22 |
| Breastfeeding, n (%) | 479 (49.59%) |
| **Children's characteristics at around age 7** |  |
| Age (year) | 6.96 ± 0.44 |
| Body height (cm) | 124.13 ± 5.00 |
| BMI (kg/m^2^) | 15.05 ± 2.29 |
| Waist-hip ratio | 0.84 ± 0.05 |
| Body fat percentage | 18.97 ± 6.98 |
| Sum of skinfold thickness (mm) | 36.06 ± 17.07 |
| Systolic BP (mmHg) | 101.88 ± 8.87 |
| Diastolic BP (mmHg) | 61.83 ± 7.90 |
| Prehypertension /hypertension, n (%) ^*^ | 142 (14.73%) |
| Carotid-femoral PWV (m/s) | 4.74 ± 0.60 |
| Fasting PG (mmol/L) | 4.58 ± 0.37 |
| 15-min PG (mmol/L) | 7.06 ± 1.19 |
| 30-min PG (mmol/L) | 7.59 ± 1.51 |
| 1-hour PG (mmol/L) | 5.93 ± 1.55 |
| 2-hour PG (mmol/L) | 5.30 ± 0.97 |
| Abnormal glucose tolerance, n (%) ^#^ | 19 (2.09%) |
| Matsuda ISI | 16.04 ± 8.91 |
| HOMA-IR at OGTT | 0.80 ± 0.76 |
| HOMA-β at OGTT | 76.94 ± 71.93 |
| IGI at 30-min | 79.32 ± 91.31 |
| TC (mmol/L) | 4.48 ± 0.73 |
| TG (mmol/L) | 0.74 ± 0.34 |
| HDL (mmol/L) | 1.66 ± 0.35 |
| LDL (mmol/L) | 2.48 ± 0.64 |
| Serum total 25(OH)D (nmol/L) | 62.49 ± 18.68 |
| Ambient solar radiation (MJ/m^2^) ^$^ | 428.17 ± 104.02 |

Continuous variables are expressed as mean ± SD and categorical variables as n (%).

^*^ Prehypertension/hypertension is defined as children’s blood pressure percentile ≥ 90^th^ percentile.

^#^ Abnormal glucose tolerance includes impaired fasting glucose (Fasting PG between 5.6 and <7.0 mmol/L), impaired glucose tolerance (2-hour PG between 7.8 and 11.0 mmol/L), and diabetes (Fasting PG ≥ 7.0 mmol/L or 2-hour PG ≥ 11.0 mmol/L).

^$^ Monthly global solar radiation recorded at the Hong Kong Observatory at the time of the oral glucose tolerance test during the study period, at delivery, and at around age 7 were obtained from the public domain.

Abbreviation: 25(OH)D, 25-hydroxyvitamin D; BMI, body mass index; BP, blood pressure; PWV, pulse wave velocity; PG, plasma glucose; ISI, insulin sensitivity index; HOMA-IR, Homeostatic Model Assessment for Insulin Resistance; HOMA-β, Homeostatic Model Assessment for β-cell function; IGI, insulinogenic index; TC, total cholesterol; TG, triglyceride; HDL, high-density lipoprotein; LDL, low-density lipoprotein; OGTT, oral glucose tolerance test.
